# Understanding the psychology of mobile gambling: A behavioural synthesis

**DOI:** 10.1111/bjop.12226

**Published:** 2016-10-18

**Authors:** Richard J. E. James, Claire O'Malley, Richard J. Tunney

**Affiliations:** ^1^ School of Psychology University of Nottingham UK; ^2^ School of Psychology University of Nottingham Malaysia Campus Semenyih Malaysia

**Keywords:** mobile gambling, gambling, behaviour, learning, problem gambling

## Abstract

This manuscript reviews the extant literature on key issues related to mobile gambling and considers whether the potential risks of harm emerging from this platform are driven by pre‐existing comorbidities or by psychological processes unique to mobile gambling. We propose an account based on associative learning that suggests this form of gambling is likely to show distinctive features compared with other gambling technologies. Smartphones are a rapidly growing platform on which individuals can gamble using specifically designed applications, adapted websites or text messaging. This review considers how mobile phone use interacts with psychological processes relevant to gambling, the games users are likely to play on smartphones, and the interactions afforded by smartphones. Our interpretation of the evidence is that the schedules of reinforcement found in gambling interact with the ways in which people tend to use smartphones that may expedite the acquisition of maladaptive learned behaviours such as problem gambling. This account is consistent with existing theories and frameworks of problem gambling and has relevance to other forms of mobile phone use.

## Background

New technologies have affected the way people work and play, and have also enabled new ways in which people can gamble. Gambling has the potential to drive changes in consumer behaviour that few forms of entertainment can. As computers entered households in the West during the 1990s, so too did the means to gamble via the Internet. Now, as smartphones become increasingly prevalent amongst the public, so too can gambling be undertaken on mobile phones. Understanding the potential risks that new technologies pose to users is important to guide health care priorities and policymaking. Identifying the risks associated with these technologies helps anticipate future stressors on practitioners and health care providers and may be instrumental in preventing some of the harm gambling entails by developing effective responsible gambling strategies and interventions for individuals having difficulties with gambling. This is especially the case as gambling is widely conceptualized as an addictive behaviour (American Psychiatric Association, 2013), with approximately 2.3% of the worldwide population meeting the criteria for problem, pathological or disordered gambling (Williams, Volberg, & Stevens, 2012), and a further subset of the population experiencing difficulties or harm associated with gambling. The prevalence of problem gambling varies across jurisdictions, depending on factors such as legislative context and methodological differences between gambling prevalence surveys. The United Kingdom tends to have lower estimates of problem gambling, between 0.4% (Wardle *et al*., 2014) and 0.9% (Wardle, Moody, Spence, *et al*., 2011), depending on the survey frame and assessment instrument used. In using the term ‘problem gambling’, this review refers both to gamblers who meet the clinical criteria for disordered gambling and to those who show significant subclinical levels of gambling harm. Problem gambling has been used in reference to multiple conceptual models in the gambling literature. The use of this term over focusing on gambling disorder is theoretically consistent with models that hypothesize that gamblers who experience harm purely due to behavioural and cognitive processes form a continuum with recreational behaviour (Blaszczynski & Nower, 2002), and research that has labelled ‘problem gamblers’ to cover significant subclinical harm (Chou & Afifi, 2011).

This review looks at mobile gambling, and whether this emerging platform has distinguishing psychological features that may be particularly risky to gamblers, either a subset of current gamblers or a new population of gamblers. This review begins by briefly discussing availability and accessibility, two factors that have previously been used to explain why certain technologies might be more harmful, and the criticisms and considerations these entail. Discussion of the issues concerning these is warranted because of the ubiquitous nature of mobile devices. Models of problem and disordered gambling frequently identify these as the initial stages in explaining how gamblers transition from recreational to disordered gambling. The issues arising from these necessitate consideration of the roles of behaviour and cognition, and how some atypical features of mobile gambling may distinguish this form of play. The review then looks at the role of associative learning and reinforcement schedules in gambling behaviour, and the role of timing. The paper finally surveys the context in which mobile gambling might be played and the types of game common in mobile gambling, with a focus on in‐play betting.

## Gambling, mobile gambling, and online gambling

Gambling is the act of playing a game or taking a risky action for money or a desired outcome (e.g., a prize). There has been a greater emphasis on the former as gambling regulators, such as The Gambling Commission in the United Kingdom, often limit regulation to real‐money gambling. Gambling has referred to the latter with prizes in lieu of money in jurisdictions where gambling is illegal (e.g., Japan), and with some forms of mobile gaming, where gambling games are played for a non‐monetary reward (Gainsbury, Hing, Delfabbro, & King, 2014; Parke, Wardle, Rigbye, & Parke, 2012).

Mobile gambling includes multiple ways in which gambling can be accessed. This can be via a bespoke app, a website optimized for mobile gambling, gambling over the phone, or via text message. Mobile gambling and mobile video gaming increasingly overlap with one another, as many free‐to‐play games include gambling games as a secondary form of play such as a mini‐game within a larger game. These typically involve users being awarded a free play on a gambling game after a certain amount of time has elapsed, offering a non‐monetary in‐game reward. Users can often purchase further plays using a secondary currency obtained within the game or real money. Although not the focus of this paper, as the status of these activities as ‘gambling’ remains uncertain in a regulatory and legislative context (The Gambling Commission, 2015), many of the considerations here will be of relevance. The online gambling literature has examined some of this under the term ‘social gambling’, which Parke *et al*. (2012) note cover a range of services that may differ considerably between websites or applications. In a briefing document produced by The Gambling Commission (2015), the UK gambling regulator refers to social gambling as covering games that include free gambling elements, and takes a ‘watching brief’ on social gaming. This is because the overwhelming majority (*c*. 85%) of social gaming users do not spend money on their app (Parke *et al*., 2012). However, The Gambling Commission note that further evidence is required as it is unclear whether there is a relationship with harmful behaviours, whether some users display signs of problem gambling‐like behaviours on these games or whether social gamblers migrate to real‐money gambling.

Gainsbury *et al*. (2014) propose a taxonomy of online gambling and games that separates different activities based on whether payment is required or optional, whether the game is chance or skilful, the platform the game is played upon and the centrality of the gambling theme to the game. In this taxonomy, ‘online gambling’ refers not only to ‘Internet gambling’ (i.e., spending money on gambling for the chance of a monetary reward) but also a wider range of activities such as social casino games, practice games, gambling video games, and competitions or tournaments based on gambling games (e.g., poker). In the context of this taxonomy, this review can be seen to examine whether the grouping Gainsbury *et al*. (2014) classifies as ‘Internet gambling’ should include a further distinction between mobile and other Internet gamblers. When mobile gambling has been discussed in research, it has often been included under the aegis of ‘Internet gambling’ (Gainsbury *et al*., 2014; Gainsbury, Wood, Russell, Hing, & Blaszczynski, 2012; Kairouz, Paradis, & Nadeau, 2011; Phillips, Ogeil, & Blaszczynski, 2012; Williams, Wood, & Parke, 2012; Yani‐de‐Soriano, Javed, & Yousafzai, 2012), without consideration given to potential differences in platform and user behaviour. Some studies have discussed wider differences, but this has not been typical of the literature (Gainsbury, 2011; Gainsbury, Liu, Russell, & Teichert, 2016). The principal concern of this review is to consider whether the way in which gamblers interact with gambling on mobile phones is broadly synonymous with other Internet gambling, or whether it has sufficiently distinctive features that might entail different considerations for individuals, practitioners, and policymakers. There is already some evidence to suggest that mobile gambling is associated with an elevated risk of problem gambling (Gainsbury *et al*., 2016), based on self‐report data from gamblers across a range of different devices.

### Online gambling

Gambling using the Internet has been viable since the mid‐1990s (Griffiths, 1999). A literature exists concerning whether Internet gambling entails a distinctive risk of problem gambling to users. Immediate explanations for this have focussed on factors such as increased availability and accessibility (Gainsbury, Wood, *et al*., 2012). Models of problem gambling commonly hypothesize that these form part of the initial step in the development of problem gambling, in which recreational gambling transitions towards mounting harm or the development of an addictive behaviour (Blaszczynski & Nower, 2002; Sharpe, 2002). From this, it follows that by making gambling more available, or shifting the landscape of the gambling environment towards games that are easier to access should entail an increase in the prevalence of problem gambling. Much of this research has relied on self‐report data to test whether Internet gamblers show a higher problem gambling prevalence (Shaffer, Peller, LaPlante, Nelson, & LaBrie, 2010), and behavioural evidence has provided mixed findings to support these predictions (LaPlante & Shaffer, 2007; Shaffer & Martin, 2011). A number of studies have concluded that Internet gambling has a higher risk of problem gambling (Griffiths, Wardle, Orford, Sproston, & Erens, 2008; McBride & Derevensky, 2009; Petry, 2006; Wood & Williams, 2007, 2011), with survey data suggesting that in relation to other forms of gambling, problem gamblers are substantially overrepresented amongst the population of Internet gamblers. It has also been argued that problem gamblers on the Internet might experience different types of harm to in‐person gamblers (Gainsbury, Russell, Hing, Wood, & Blaszczynski, 2013). These findings have three important caveats that have queried whether Internet gambling poses a direct causal risk for problem gambling, but instead forms part of a constellation of risk factors found in high‐frequency gamblers (Gainsbury, 2015).

The first challenges the nature of the association between availability or accessibility and problem gambling. LaPlante and Shaffer (2007) studied data from a combination of gambling prevalence surveys, regional estimates of exposure, longitudinal research, and self‐exclusion rates to examine whether populations adapt to changing circumstances. These circumstances might include the implementation of liberalizing gambling legislation or an increase in the number of opportunities to gamble. They found there was an increase in the prevalence of problem gambling in the short and medium term, but not the long term suggesting support for an adaptation hypothesis where the risk of problem gambling attenuates over time. More generally, they concluded that the relationship between these environmental factors and problem gambling was related to other social factors rather than a direct relationship. However, further research has suggested that the ability of gamblers to adapt to changing circumstance depends upon their involvement with gambling. LaPlante, Schumann, LaBrie, and Shaffer (2008) found that adaptation differed as a function of involvement, with more involved gamblers showing less adaptation to novel gambling (i.e., did not show a reduction in gambling) amongst a sample of gamblers in the period shortly after they subscribed to an online betting website.

There is also the question of what is meant by availability: LaPlante and Shaffer (2007) primarily considered availability on a population‐wide level. Multiple studies have also looked at the link between geographic proximity of gambling establishments and problem gambling. These found that individuals living closer to casinos have a greater risk of problem gambling, and the density of casinos is positively associated with the risk of problem gambling (Slutske, Deutsch, Statham, & Martin, 2015; St‐Pierre, Walker, Derevensky, & Gupta, 2014; Welte, Barnes, Tidwell, Hoffman, & Wieczorek, 2016). Similar findings have been identified with fixed odds betting terminals in bookmakers, which are the UK analogue of electronic gaming machines in other jurisdictions (Wardle, Keily, Astbury, & Reith, 2012). Further studies have found that modelling for electronic gaming machine density removes most of the effect of availability on gambling and problem gambling (Slutske *et al*., 2015). In the wider addiction literature, there is a clear behavioural rationale that incidental environmental cues are associated with the activation of drug‐based addictive behaviours (Crombag, Bossert, Koya, & Shaham, 2008; Hogarth, Dickinson, & Duka, 2009). For example, many individuals with a substance use disorder experience feelings of craving in locations where they previously purchased or used a drug. However, the relationship between availability and Internet gambling is unclear. The means to gamble (i.e., an Internet connected device) is ubiquitously available, more so than any other form of gambling, yet population‐wide engagement in jurisdictions where there are few restrictions to Internet gambling is relatively low (Wardle, Moody, Griffiths, Orford, & Volberg, 2011). This is despite several forms of Internet gambling being embedded in the public consciousness (e.g., online poker). The semi‐permanence of gambling‐related cues, such as the presence of a bookmaker or casino, might be more salient than an online advert or email that can be closed or deleted at will.

The second is that comparisons of problem gambling prevalence between Internet and non‐Internet gamblers have generally failed to consider the importance of involvement, and analyses that adjust for this have tended not to demonstrate similar effects. It has been argued this might be due to the methodological approaches typically used in the Internet gambling literature. Shaffer *et al*. (2010) found that prior Internet gambling research, in a systematic search of the literature, was either primarily commentary, or the data collected was self‐report/survey data. This led them to call for further research using behavioural data from Internet gamblers, of which several analyses have been conducted before and since (e.g., Braverman, LaBrie, & Shaffer, 2011; Gray, LaPlante, & Shaffer, 2012; Xuan & Shaffer, 2009). Although problem gambling prevalence is higher amongst gamblers who play on the Internet, it is argued that this might be because these gamblers are seeking as many means to gamble as possible, and so is a consequence of harmful play in a multitude of contexts and environments rather than being caused by Internet gambling. Studies that have attempted to control for involvement have generally failed to find an increased risk of gambling problems amongst Internet gamblers (Afifi, LaPlante, Taillieu, Dowd, & Shaffer, 2014; LaPlante, Nelson, & Gray, 2014). In a similar vein, studies using survey data that compared Internet, retail, and mixed gamblers found that risks of problem gambling were present in mixed use but not online‐only gamblers suggesting again the role of involvement in online gambling harm (Gainsbury, Russell, Blaszczynski, & Hing, 2015; Wardle, Moody, Griffiths, *et al*., 2011). Latent class analyses of gamblers and Internet gamblers specifically (Lloyd *et al*., 2010; Wardle *et al*., 2014) have strongly suggested that there several different subtypes of gambler on the basis of the games they play, and that increased risk measured by Internet gambling studies in fact comprise a group of multimodal, multi‐game gamblers. Along similar lines, studies comparing subtypes of problem gambling derived from latent class analysis found that intermediate and high severity gamblers did not differ their probability of engaging in Internet gambling with no difference in Internet sports betting, an activity that is pertinent to mobile gambling (James, O'Malley, & Tunney, 2016a).

The third consideration is that is unclear that the structural features of Internet gambling are particularly different from forms of play in a bookmaker or casino, which might explain why the relationship between Internet and problem gambling is mixed. Most contemporary gaming machines are computerized, and so are likely to have similar software to that running on an Internet gambling site, attenuating the behavioural differences between the two types of gambling (Floyd, Whelan, & Meyers, 2006). Differences between the two are therefore likely to focus more on contextual factors or the medium on which it is delivered. On this, recent commentaries in the field of ‘Internet addiction’ cast doubt on the latter, arguing that the addictiveness of the Internet as a medium is conceptually unsound (Starcevic, 2013). However, it has been speculated that in some cases, the use of the Internet might moderate the relationship between the individual and a potentially addictive behaviour (Starcevic & Aboujaoude, 2017).

In summary, it is clear that Internet gamblers are overrepresented amongst problem gamblers and there is a basis to suggest the same might occur with mobile gambling. What is unclear is why: Is it because the means to do so are highly available, in an environment that is more likely than not to leave gamblers isolated? Alternatively, studies that have attempted to control for gamblers’ levels of involvement with gambling have found some types of game (EGM and ‘live action’ or ‘in‐play’ gambling) are associated with problems but not Internet gambling as a whole (Afifi *et al*., 2014; Gray *et al*., 2012; Hing, Russell, Vitartas, & Lamont, 2015). This perspective implies problem gamblers diversify the range of games they play in, and Internet gambling is one means amongst many; given the relatively low uptake of Internet gambling, this suggests the risk associated with Internet gambling is not distinctive from other forms of play. Regardless, while accessibility and involvement are important components in gambling and problem gambling, and the near constant presence of mobile phones suggests this is an important area to consider as mobile gambling grows, it is at present unlikely to provide a more satisfying answer than the present literature on Internet gambling. It is therefore of greater utility to look at the next stage, the role of behaviour.

## Behavioural mechanisms

Gambling is a behaviour that operates on a ‘random ratio’ (RR) schedule of reinforcement; this means the desired reinforcer (e.g., winning, money, physiological arousal) occurs on average after a pre‐specified number of gambles, but that the number of intervening trials between wins may vary, such as in the fixed‐odds scenarios that comprise many games of chance. The random ratio is similar to the variable ratio schedule of reinforcement. This schedule of reinforcement has long been demonstrated to rapidly produce a frequent level of gambling that is difficult to suppress (Dickerson, 1984; Skinner, 1972) and has been found to take longer to extinguish in high‐frequency gamblers (Horsley, Osborne, Norman, & Wells, 2012), showing deficits in partial reinforcement that demonstrate themselves in greater perseverative gambling not unlike loss‐chasing. There is some evidence that longer delays between gambles contributes to continued play, in the form of lottery games (Griffiths & Auer, 2013) – gambling prevalence research has consistently found that lottery games are amongst the most popular with the general public (Sproston, Erens, & Orford, 2000; Wardle, Moody, Spence, *et al*., 2011) and often have large latencies between gambles.

This schedule of reinforcement appears to be particularly relevant for certain types of game, such as slot machines, electronic gaming machines and fixed odds betting terminals. In addition, research in betting has identified the importance of timing in the form of the fixed interval (FI) schedule. Dickerson (1979) noted that a ‘late betting’ effect was observed in high‐frequency gamblers. This was interpreted in terms of physiological arousal, which is a core element of cognitive‐behavioural approaches to problem gambling (Coventry & Brown, 1993; Sharpe, 2002). In addition to being present on a FI basis, physiological arousal is also present in a more frequent RR schedule, partially independent of winning outcomes in the form of a near miss (Reid, 1986) or losses disguised as wins (Dixon, Harrigan, Sandhu, Collins, & Fugelsang, 2010). These both produce high levels of arousal that appear to stimulate continued gambling. These have been typically studied in simulated slot machine games as the sequential stopping of slot reels produces strong feelings of anticipation. Economic analyses of online betting data in Italy, although not considering a behavioural explanation, appeared to find a similar effect to late betting with data from over a million bets; performance was worse when bets were made closer to the beginning of an event (Innocenti, Nannicini, & Ricciuti, 2014). Theories of problem gambling such as the pathways model (Blaszczynski & Nower, 2002) claim that extensive exposure to these processes and the development of maladaptive conditioned behaviours and cognitive biases underpin the transition between recreational and problem gambling.

The literature investigating smartphone app use has suggested that mobile phone users engage with their device in a manner that may be conductive to the conditioning of habitual or problematic behaviours. Mobile phone users engage with apps in a fairly consistent manner; using a relatively limited range of apps on a very frequent basis. Most users download apps on a frequent basis although this varies by age (Ofcom, 2014), use a moderate range of these on a quarterly basis (The Nielsen Company, 2014b), and much more restricted number of these on a regular basis (The Nielsen Company, 2014b; Walker, 2012). The way in which these apps are used once downloaded appears to be similar across users. Studies have demonstrated that users engage with mobile phone apps in excess of 1 hr per day (Bohmer, Hecht, Schoning, Kruger, & Bauer, 2011) and is increasing (The Nielsen Company, 2014a), but only use these apps for approximately 1–2 min per session (Bohmer *et al*., 2011; Tossell, Kortum, Rahmati, Shepard, & Zhong, 2012). Furthermore, in using applications over time, the behaviour appears to be habitual or ‘checking’ in nature (Oulasvirta, Rattenbury, Ma, & Raita, 2012). Much has been made of this finding in regard to the potential for harmful mobile phone related behaviours (Lee, Chang, Lin, & Cheng, 2014; van Deursen, Bolle, Hegner, & Kommers, 2015). These checking behaviours generally focussed on a single application, but this was associated with engagement with other apps on their phone, such that users engaged with sequences of apps in a regular fashion. Combined, this suggests that users engage with a small set of apps on a frequent basis, on which users will regularly play for a small period of time many times a day.

One of the central features of mobile app use in general is the role of intermittent periods of engagement with an app. Mobile phone users interact with their phone on a frequent, habitual, and intermittent basis (Oulasvirta *et al*., 2012). Such a schedule of reinforcement in the context of gambling has the potential for the development of harmful behaviours. In the associative learning literature, there is a body of research on the effects of inter trial interval, or the gap between two reinforcements, on learned behaviours (Barela, 1999; Bouton, Woods, & Todd, 2014; Gallistel & Gibbon, 2000; Moody, Sunsay, & Bouton, 2006; Sunsay & Bouton, 2008), which suggests that distinct psychological processes might contribute to mobile gambling. This research has amply demonstrated that longer intermissions between reinforcing events (i.e., gambles, wins) produces faster acquisition of conditioned behaviours. The role of these ‘snacking’‐like behaviours in mobile gambling is that a ‘snack’ like orintermittent schedule of reinforcement might lead to users acquiring gambling behaviours (including harmful behaviours if contemporary models of problem gambling are supported) more rapidly than other forms of gambling. It is presently disputed whether this also affects the suppression or extinction of learned behaviours (Bouton *et al*., 2014; Gallistel & Gibbon, 2000) in the same manner, although there is increasing evidence to support this (Bouton *et al*., 2014; Moody *et al*., 2006). There is already evidence within the gambling literature to suggest that this prediction is already partially realized; Blaszczynski, Cowley, Anthony, and Hinsley (2015) found that craving to gamble increased in line with intersession interval on a simulated slot machine game. While they provided an explanation based on theories of behavioural completion, this finding can be adequately described with an associative learning‐based account. This stands in contrast with a wider literature on breaks in play, although Blaszczynski *et al*. (2015) note these include additional interventions that require gamblers to think about their play and it may be the content of these messages that drive reappraisal of gambling behaviour. Similarly, James, O'Malley, and Tunney (2016b), in studying the role of inter trial intervals in gambling behaviour, found that perseverative gambling during extinction in a simulated slot machine game was affected by the amount of inter trial interval participants were exposed to; longer intertrial intervals were associated with gambling in the face of continued losses, particularly at lower rates of reinforcement. The implications of this are clear. Given that associative processes are thought to be instrumental in the development of problem gambling, this suggests that the acquisition of harmful gambling behaviours will be accelerated in mobile gamblers relative to other gamblers. This strongly suggests there is reason to identify mobile gambling as separate from other interactive gambling technologies.

This also has important qualifications for many responsible gambling interventions. Many of these approaches or interventions aim to reduce problematic gambling behaviour by breaking up individuals’ play alongside messages about the risks of gambling. It might be the case that further consideration ought to be taken in tailoring responsible gambling strategies, particularly with a technology where typical user behaviour and often (especially in the case of video games) the developer's intention is to force latencies between uses to extend play. It may be the case that current responsible gambling strategies may be less efficacious with mobile gambling technologies.

In addition to the behavioural processes maintaining and reinforcing gambling behaviour, there are mechanisms governing the distribution of responses to different forms of gambling play. One example of this is the matching law (Herrnstein, 1974) and its generalization (Baum, 1974), which attempts to describe how organisms distribute responding to multiple concurrent ratio or interval schedules. There is a literature on response allocation in concurrent slot machines, but findings in this area have been mixed; a number of studies (Coates & Blaszczynski, 2014; Daly *et al*., 2014; Dixon, Fugelsang, MacLaren, & Harrigan, 2013; Dixon, MacLin, & Daugherty, 2006; Dymond, McCann, Griffiths, Cox, & Crocker, 2012; Zlomke & Dixon, 2006) found evidence consistent with matching, but there is also evidence gamblers undermatch, showing greater (or in some cases, total) equivalence between machines that diverge either in rate of return to player or rate of reinforcement on a ratio schedule (Coates & Blaszczynski, 2013; Daly *et al*., 2014; Lucas & Singh, 2012; Weatherly, Thompson, Hodny, Meier, & Dixon, 2009). In addition, matching is highly susceptible to being overridden by contextual cues (Nastally, Dixon, & Jackson, 2010; Zlomke & Dixon, 2006) although this appears to weaken with extended exposure to the contingencies of a machine (Hoon & Dymond, 2013). Furthermore, there are some situations such as on multiple line slot machines where the rate of reinforcement can be (and is) controlled by the player while the rate of return remains the same (MacLaren, 2015).

There have also been analyses of pools betting that suggest in betting on the outcome of college basketball games that people probability match, making predictions based on past frequencies, and overestimating the probability of upsets (McCrea & Hirt, 2009). This pattern of behaviour, specifically a greater resistance towards maximizing when asked to predict a guaranteed outcome between two choices with different rates of reinforcement, has been found to be more common amongst problem gamblers (Gaissmaier, Wilke, Scheibehenne, McCanney, & Barrett, 2016). Although frequently attributed to the matching law, this is actually a violation of this principle; when presented with a choice where an outcome is guaranteed, the matching law predicts the selection of the choice with the highest rate of reinforcement (Herrnstein & Loveland, 1975; Shanks, Tunney, & McCarthy, 2002). While evidence on this is sparse, this may be common to a number of different types of betting behaviour, not just pools but accumulator and more standard betting. Adherence and divergence from the matching law may be one of the factors that separates betting from games of chance. As discussed earlier, a consensus has emerged in the Internet gambling literature that broadly suggests the importance of involvement rather than any specific effect of the platform, the type of games played online or availability/accessibility. The behavioural processes outlined in this section cannot be readily explained by involvement as these affect a different stage of the transition from recreational to problematic gambling as predicted by contemporary models (Blaszczynski & Nower, 2002; Sharpe, 2002). The remainder of this review will outline the context in which mobile gambling is played, focusing on the sensing capabilities of smartphones versus other remote gambling hardware, where mobile gambling is played, the games that are played and the restrictions that are placed on accessing mobile gambling.

## Mobile gambling behaviours

Smartphones allow a greater range of interactions with the user than other computers. These can be used to deliver a unique gambling experience over and above other online gambling. Until these more recent generations of smartphone, the graphical and processing limitations of mobile phones meant that the rich gambling environments necessary for some types of gaming were not possible (Griffiths, 2007). The range of sensors included in most contemporary smartphones (alongside more sophisticated hardware) offers the possibility of a personalized gambling experience that is more enjoyable and interactive than traditional online offerings. However, business analysis of the remote gambling market suggested there were gaps in user friendliness and experience that prevented this from being realized (Pietkanien, 2014). This means that while the mobile gambling experience can differ from online and Internet gambling, this appears to remain a potential difference. However, it is possible future growth in the mobile gambling market may be driven by applications that take advantage of these, driving further differences between mobile and Internet gambling.

### Context of use

Internet gambling is much more constrained in the context in which a device can be used than mobile gambling. This is illustrated when considering the advertisements that are used to promote gambling apps, although rigorous research on the content of gambling advertising in the United Kingdom is relatively limited (Binde, 2014). Many of these are presented in social environments, such as at pubs or as a complement to sporting events, either during sports programmes or at the event itself (Parke, Harris, Parke, Rigbye, & Blaszczynski, 2014). Unlike other gambling technologies, mobile gambling allows users to gamble at these locations. Other literature that has considered mobile gambling has suggested that it may be engaged with as an adjunct to everyday activities, such as travelling or watching television. Griffiths (2007) notes that mobile gambling occurs in different contexts to online gambling, and in contexts that are more amenable to gambling, which suggests that mobile gambling might be a more enjoyable experience. Indicators from gambling operators and consultants (Ladbrokes, 2015; Pietkanien, 2014) suggest that the operators are finding that while shop and mobile betting do not appear to overlap at present, this does not necessarily appear to be the case between desktop and mobile gambling. An obvious explanation for this is that the context in which mobile gambling can be engaged is more similar to in‐person gambling and is less constrained by having to be on a computer and so users are migrating from desktop to smartphones. In contrast, the research on online gambling conducted as the first and second generations of smartphones came on the market indicated that the vast majority of users gambled from home (97%), with very little engagement in other locations (McBride & Derevensky, 2009). The other prevailing responses, all engaged in by <15% of users, primarily focus on using PCs in other locations (e.g., at work). It should be noted that this did include mobile phones, which 2.3% of the sample had used to gamble. Recent data from The Gambling Commission (2016) suggest that while the most common place to gamble on the Internet is at home (97% of gamblers played at home), younger gamblers (<35 s) are increasingly gambling while commuting, at sports events or in social environments (e.g., pubs). Context of use is also important when contrasting mobile and retail gambling as one of the potentially attractive features of both mobile and online gambling is the private nature of online/mobile gambling, and that retail gambling locations may have a tendency to discourage some potential gamblers because of the negative societal connotations associated with them (Gainsbury, Wood, *et al*., 2012).

Another reason why mobile and retail gambling operations may not overlap is the demographic profile of mobile gamblers. Comments from gambling industry executives to the House of Commons Culture Media & Sport Select Committee (2012) in the United Kingdom indicated mobile gambling operators believe that mobile gamblers are younger and may not previously have interacted with gambling before. Similarly, Gainsbury, Russell, and Blaszczynski (2012) found that university students were more likely to gamble using a smartphone. This has until recently been borne out in the demographic profiles of smartphone owners (Ofcom, 2015), but this is now changing. The attraction of mobile gambling to this audience is also relevant to its relationship with problem gambling, as problem gambling is more common in younger gamblers despite a lower prevalence of gambling (Wardle, Moody, Spence, *et al*., 2011).

### Types of games

Mobile gambling has traditionally had a heavier emphasis on sports betting than other forms of gambling (Griffiths, 2007). However, there is evidence that the mobile market is changing, with the annual reports of major UK gambling operators reporting increased investment in casino style games as mobile technologies allow an aesthetic experience similar to other Internet gambling (Ladbrokes, 2015; William Hill, 2015). Betting remains the main source of revenue for operators and this is continuing to increase; the 2014 World Cup was heralded as a ‘mobile tournament’ operators in the United Kingdom as gamblers increasingly used their mobile phones to wager (Ladbrokes, 2015), likely helped in part by the evening kick‐off of many games.

There is limited data on the types of games played on mobile. Much of the data concerns ‘remote’ gambling, a composite term for all Internet gambling. However, from comparing what evidence is available, there are some broad trends that can be gleaned. A report by H2 Capital (2013) indicates that the majority of online gambling (defined by gross win) comprises online sports betting, making up just over 50% of the market. However, a report commissioned by HM Revenues & Customs (Frontier Economics, 2014) suggests that remote gaming (i.e., casino games) rather than betting makes up the majority of revenue in the UK market. Similarly, data from a report on online gambling in the European internal market (The European Commission, 2012) show that while betting enjoys a plurality of market share (32%) in the largest legal market for online gambling, it is closely followed by casino gaming (22%) and poker (21%). For mobile gambling, figures from the major UK operators where show a very strong bias towards betting. In the annual reports and financial returns of these companies, the proportion of revenues obtained from sports and other betting exceed 60% of total mobile profit. However, it should be noted that for the major operators for which data are available (Betfair, 2015; Ladbrokes, 2015; Paddy Power, 2015; William Hill, 2015), all bar one of these are major retail bookmakers in the United Kingdom (the other is a betting market). These also report some of their fastest increases in revenue for their mobile casino operations. While betting appears to be the predominant form of mobile gambling, there appears to be notable growth in casino style games.

UK operators frequently advertise mobile apps alongside in‐play betting, a form of betting where wagers can be made on various outcomes during a sporting event, and typically where the odds rapidly change over relatively short periods of time. It is important to note that marketing of mobile gambling frequently presents in‐play gambling as a normative mobile gambling activity. The effect of gambling advertising on attitudes and behaviour has been well recognized (Binde, 2014; Derevensky, Sklar, Gupta, & Messerlian, 2009; Parke *et al*., 2014), and in‐play (or ‘live action’) betting is an activity that is known to have an increased risk of harm. Research on in‐play betting has identified this form of gambling as being a particular risk factor for problem gambling behaviours (Brosowski, Meyer, & Hayer, 2012). LaPlante *et al*. (2014) analysed data from European Internet gamblers, finding that use of in‐play betting was associated with problematic and harmful behaviour when controlling for involvement. However, this also highlights that in‐play betting is available on Internet gambling websites as well as mobile phones. The causal mechanism behind this association with problem gambling is unknown, and it has been speculated that either the potentially continuous schedule of gambling or the shorter delay between wager, outcome, and reward might drive this risk. It is also unclear whether in‐play, like mobile or online, has a causal link with problem gambling, or whether it is particularly attractive to individuals who are problem gamblers or are prone to developing addictive behaviours. Behaviourally in‐play offers a large array of opportunities to gamble within a single sporting event, alongside a highly variable rate of reinforcement. Given in‐play bettors showed a lower net loss than other forms of betting in the European betting site data, this might be due to in‐play having a higher win rate, or the success of lower odds bets. The former might indicate that in‐play gambling encourages players, particularly gamblers transitioning from other forms of gambling, to ‘accelerate’ their responding (i.e., by gambling more) in line with the law of effect (Herrnstein, 1970). Alternatively, models of addiction and problem gambling in reinforcement learning highlight how statistically unexpected wins are likely to create a ‘state‐splitting’ effect that would lead to gambling that is very difficult to extinguish (Redish, Jensen, Johnson, & Kurth‐Nelson, 2007). Although there is an association between this form of play, prevalent on mobile phones, and problem gambling when controlling for involvement, in‐depth research on in‐play betting is sparse.

Unlike Internet gambling, it is easier to restrict mobile gambling, particularly via app use. Because the majority of apps are downloaded via two app stores, and these can restrict content based on location, it is more difficult to circumvent restrictions on gambling apps than a PC or laptop. As an example of legal restrictions, gambling apps are restricted in America as online gambling is severely curtailed following the Unlawful Internet Gambling Enforcement Act 2006, and so most are unavailable on the US version of the iOS App Store (social gambling games are available). Furthermore, the availability of gambling apps on Android phones is more limited than iOS as these apps are banned on the Google Play Store. However, gambling apps can still be installed onto devices, and some major UK operators have Android offerings. However, given the potential role of availability, this restriction may be of considerable importance. Google Play does allow free‐to‐play casino gambling apps on their store, in which further credits or other items can be bought with real‐money in‐game, but do not award real money. The Apple App Store allows real‐money gambling (although the app must be free to purchase) for betting, casino, and other gambling games in a number of jurisdictions, including the United Kingdom, Ireland, and Australia. Other apps differ by jurisdiction. For instance, major betting operators have a different app to download in Australia where in‐play betting is currently restricted (William Hill, 2015).

## Conclusion

The evidence on Internet gambling has suggested that the associated risks with this form of play often focus on highly engaged gamblers, and that the wide range of games that are offered appear to be harmful to these gamblers. Mobile gambling represents a potential vector for future and further harms as the interactions that characterize mobile phone use alongside gambling's schedules of reinforcement suggest that mobile gambling will accelerate the acquisition of gambling‐related associations. In addition, some games promoted on smartphones are associated with problem gambling‐related harms, even when controlling for involvement. This review identifies several potential differences between mobile and other Internet gambling that may be cause for concern. Mobile app use engenders a consistent pattern of behaviour that has been previously described as habitual. Mobile devices are used in different contexts and more often than not different games are played on them to other online gambling, although increasing convergence between online and mobile games in the broadest sense might occur in the future. Mobile games offer the potential for a user experience quite different from online games as they include a wider range of sensors that can be used to personalize gambling in a way distinct from Internet and other gambling. Although research to address this is required, the evidence presented here indicates there are several sources of differentiation between mobile and other Internet gambling, and that the psychological implications of these differences on gamblers potentially pose important questions that ought to be addressed as mobile gambling continues to be adopted by users.

## Financial support

This work was supported by the Economic and Social Research Council (ES/J500100/1) and the Engineering and Physical Sciences Research Council (EP/G037574/1).
